# Orbital Apex Syndrome Secondary to Huge Primary Ethmoidal Sinus Mucocele: A Case Report

**DOI:** 10.7759/cureus.34853

**Published:** 2023-02-11

**Authors:** Sue Anne Loh, Wan-Hazabbah Wan Hitam, Ramiza Ramza Ramli, Khairil Amir Sayuti, Khairy Shamel Sonny Teo

**Affiliations:** 1 Department of Ophthalmology and Visual Science, School of Medical Sciences, Health Campus, Universiti Sains Malaysia, Kelantan, MYS; 2 Department of Otorhinolaryngology-Head and Neck Surgery, School of Medical Sciences, Health Campus, Universiti Sains Malaysia, Kelantan, MYS; 3 Department of Radiology, School of Medical Sciences, Health Campus, Universiti Sains Malaysia, Kelantan, MYS

**Keywords:** mucocele, ethmoidal mucocele, marsupialization, endoscopic sinus surgery, orbital apex syndrome

## Abstract

Ethmoidal sinus mucoceles are benign expansile lesions that may progressively invade the orbit causing optic nerve compression and its nearby structures. We report a rare case of primary ethmoidal sinus mucocele instigating orbital apex syndrome. A 40-year-old man presented with right eye (RE) progressive blurring of vision with diplopia for 2 weeks. It was preceded by right-sided facial pain for 3 months. Clinical examination revealed RE proptosis with multiple cranial nerves palsy involving right cranial nerves II, III, IV, V, and VI, suggestive of right orbital apex syndrome. Magnetic resonance imaging (MRI) demonstrated right eye proptosis and right ethmoidal mucocele with intracranial and right intraorbital extension compressing the right medial rectus and optic nerve. The patient underwent an uncomplicated endoscopic sinus surgery resulting in a return to normal appearance and function post-operation. Thus, ethmoidal mucoceles are benign and curable with early recognition and intervention.

## Introduction

Paranasal sinus mucocele is a benign cystic lesion caused by obstruction of sinus ostia [1]. This locally expansile mass is highly likely to invade the intraorbital or intracranial structures due to its proximity to the orbit and skull base, with possible adverse complications and morbidities [2]. The wide range of ophthalmic manifestations includes proptosis, limitation of ocular movement, diplopia, lid swelling, ptosis, reduced vision, visual field defect, epiphora, and periorbital pain [3]. It is rare for an ethmoidal sinus mucocele to cause orbital apex syndrome without obvious predisposing factors. Hence, we report a rare case of orbital apex syndrome secondary to the primary ethmoidal mucocele that was successfully treated.

## Case presentation

A 40-year-old man, without any comorbidity, presented with progressive painless proptosis of the right eye (RE) for the past two years. Subsequently, he had right-sided facial pain for the past three months and started to have blurred vision and diplopia for the past two weeks. Further, no history of eye redness, swelling, or discharge was recorded. The patient also did not experience any nasal obstruction, epistaxis, fever, headache, dizziness, nausea, or vomiting. He had no history of trauma, head and neck disease, or surgery. His premorbid vision was good and he was not prescribed any glasses.

**Figure 1:**
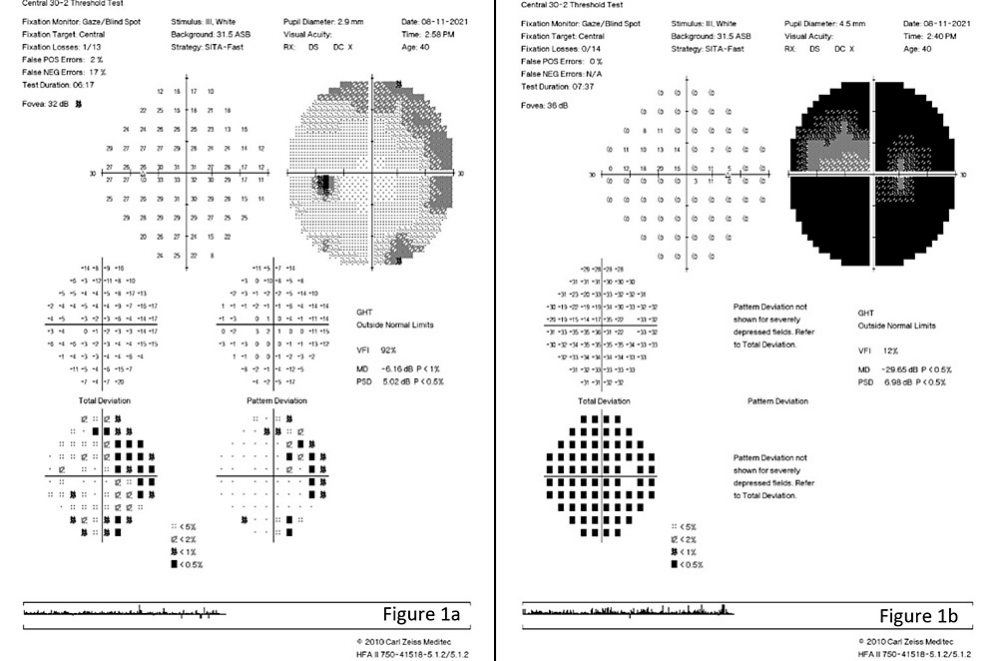
Humphrey visual field showed LE showed superonasal defect (a) and RE severely depressed fields (b). LE: left eye; RE: right eye

**Figure 2:**
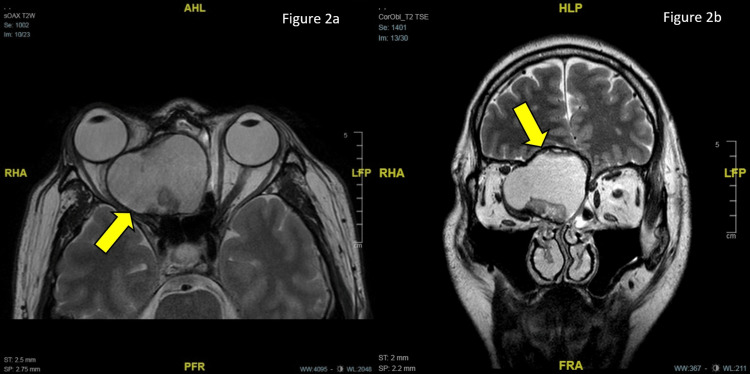
Preoperative axial (a) and coronal (b) T2-weighted MRI image of the brain showing hyperintense lesion in the ethmoidal sinuses with intracranial and right intraorbital extension, right eye proptosis as well as mass effect affecting the right orbital apex.

On examination, there was non-axial proptosis of the RE measuring 22 mm and of the left eye (LE) measuring 15 mm, measured with a Hertel exophthalmometer. His best corrected visual acuity (BCVA) was 6/15 in the RE and 6/6 in his LE. His RE extraocular motility is slightly limited in adduction, abduction, and elevation with diplopia on horizontal gazes. There was a positive right relative afferent pupillary defect with reduced light brightness, red saturation, and color vision on Ishihara charting. Both anterior segments were unremarkable. Fundoscopy showed a normal optic disc and macula in both eyes. Humphrey’s visual field showed superonasal defect over LE (Figure 1a) and severely depressed fields over RE (Mean deviation -29.65dB) (Figure 1b). MRI revealed a right ethmoidal mucocele with intracranial and right intraorbital extension compressing the right medial rectus and optic nerve as well as RE proptosis (Figure 2).

**Figure 3:**
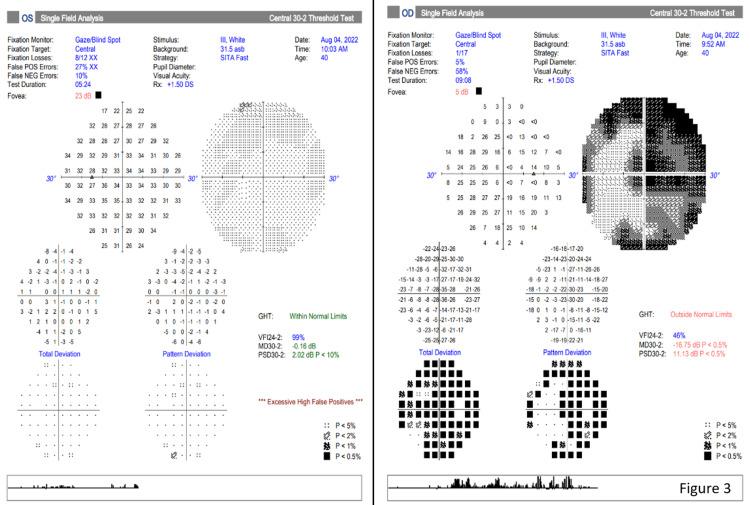
Humphrey visual field showed BE improved visual field. BE: bilateral eye

**Figure 4:**
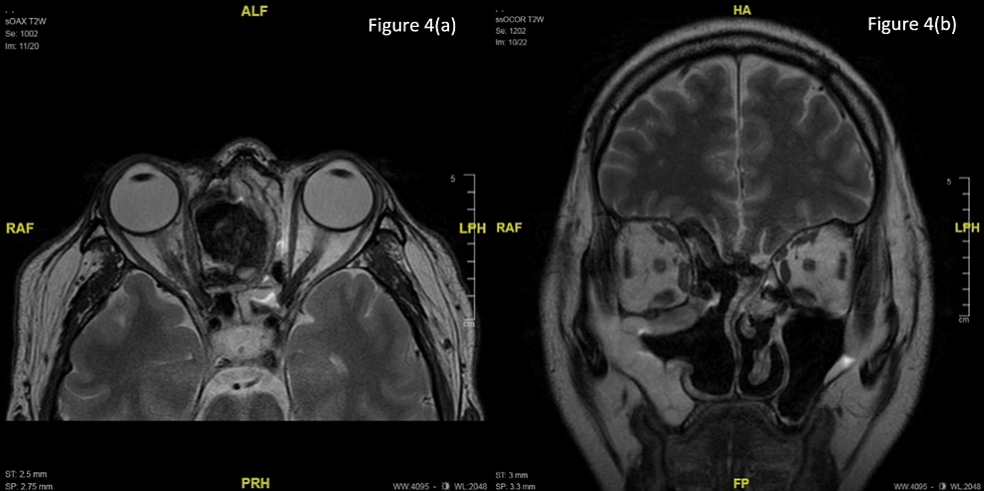
Postoperative axial (a) and coronal (b) T2-weighted MRI image of the brain showing no residual lesion with the resolution of right eye proptosis with symmetrical optic nerves and preservation of extraocular muscles.

The patient was diagnosed with orbital apex syndrome secondary to ethmoidal mucocele and referred to the otorhinolaryngology team for surgical management to prevent further optic nerve damage and visual acuity deterioration. As part of treatment, an endoscopic marsupialization of the right ethmoidal mucocele with partial medial maxillectomy and right inferior and middle turbinectomies was performed. Intraoperatively, an incision was made at the inferior part of the mucocele and peanut butter-like mucus content was drained. Histopathology examination of the aspirate samples and tissues revealed mucocele content comprising fibro-collagenous tissues lined by respiratory epithelium with chronic inflammatory cells and granulation tissue. Postoperatively, he underwent a successful recovery with complete resolution of the proptosis and is still under our follow-up. Eight months of recovery period post-operation showed normal extraocular movement and no diplopia. Optic nerve function tests were also improved. His both eyes BCVA were 6/6 with normal fundoscopy findings. The bilateral eye (BE)visual field improved as evidenced by Humphrey’s visual field of a normal LE visual field and RE central vision (Figure 3). Postoperative MRI brain showed no residual lesion (Figure 4).

## Discussion

Mucoceles can either be primary lesions presenting as mucus retention cysts or secondary lesions caused by various conditions including chronic obstruction of sinus Ostia, previous surgical procedures, mucosal inflammation, benign and malignant lesions, chronic infection, or allergic disease [4]. The most common sites for paranasal sinus mucocele are the maxillary sinuses (50%) followed by frontoethmoidal (31%), ethmoidal (16%), and sphenoidal (3%) sinuses [5].

Primary mucoceles are seldom reported but are typically found in ethmoidal sinuses like in our patient [1]. The unusual thing about our case is that the patient reported no previous sinus issues or previous surgery. The ophthalmological manifestations may be variable depending on the location and size of the mucocele, involvement of adjacent tissues, and direction of expansion [6]. In general, frontal and anterior ethmoid sinus mucocele tend to cause proptosis, eyeball displacement, and diplopia consistent with the presentation of our patient [7]. However, our patient also had orbital apex syndrome suggested by reduced visual acuity, ophthalmoplegia, diplopia, and facial pain which are usually caused by sphenoid rather than ethmoidal mucoceles.

MRI is crucial for mucocele diagnosis, determination of lesion location, and assessment of possible intraorbital and intracranial extension. MRI signal intensity is highly variable and depends on the proportion of water, mucus viscosity, and protein content of the tissues [8]. The scan is also important to help differentiate mucoceles from other lesions. Mucoceles characteristically reveal a thin peripheral linear enhancement with central low-signal intensity on T1-weighted images. Differential diagnoses include paranasal sinus tumors (mostly show diffuse contrast enhancement), mucus retention cyst (does not fill the sinus and no bony expansion), antrochoanal polyp (focally protrudes through the ostiomeatal complex), acute sinusitis (no bony expansion) and *Aspergillus *sp. Infection (displays a low signal on both T1- and T2-weighted sequences, mimicking a normal aerated sinus). Previous studies revealed that the presence of air within an affected sinus rules out the possibility of a mucocele [9].

Surgical intervention remains the gold standard of mucocele treatment. Ethmoidal mucoceles were conventionally treated with an external open obliterative procedure [10]. Otherwise, endoscopic surgery can also be performed. The endoscopic marsupialization approach is preferred over the external approach as it has been reported to have lower complications, short recovery time, low recurrence rate, and morbidity [10]. However, in cases with extensive intraorbital extensions, as in our patient, open or combined approaches are recommended [2]. The external approach may directly expose the entire sinus, enable complete sinus removal, and prevent blind curettage of any exposed dura mater. Our patient had an extensive mass (3.9 x 4.9 x 3.8 cm) that affected the mid and posterior orbit and intracranial extension, but he was successfully treated via the endoscopic approach. Postoperatively, he has no residual mass on MRI and demonstrated resolution of symptoms. Thus, endoscopy could be a favorable approach for an extensive mass.

The histopathological appearance of mucoceles has features of respiratory mucosa, lined by flattened, ciliated mucus-secreting columnar epithelium. In some cases, there may be areas of reactive bone formation adjacent to the epithelium. Chronic inflammation is often present with mucinous material. Rupture may cause granulation tissue, fibrosis, cholesterol granuloma, or cleft formation [6]. The natural development of ethmoidal mucoceles is a gradual expansion, which may become erosive and destroy the surrounding bony wall. This may cause a variety of complications such as meningitis, meningoencephalitis, brain abscess, seizures, and cerebrospinal fluid fistulas. However, the prognosis remains good and it has a low recurrence rate if it is managed early and supported with long-term follow-up [11].

## Conclusions

Although benign and rare, ethmoidal sinus mucoceles may lead to irreversible blindness due to the compression of the optic nerve and its adjacent structures. An early surgical intervention mainly via endoscopic sinus surgery may prevent permanent visual loss.
